# SmartPhase: Accurate and fast phasing of heterozygous variant pairs for genetic diagnosis of rare diseases

**DOI:** 10.1371/journal.pcbi.1007613

**Published:** 2020-02-07

**Authors:** Paul Hager, Hans-Werner Mewes, Meino Rohlfs, Christoph Klein, Tim Jeske

**Affiliations:** 1 Institute of Bioinformatics and Systems Biology, Helmholtz Zentrum München GmbH, Neuherberg, Germany; 2 Technische Universität München, School of Life Sciences, Weihenstephan, Freising, Germany; 3 Department of Pediatrics, Dr. von Hauner Children’s Hospital, University Hospital, LMU Munich, München, Germany; Johns Hopkins University, UNITED STATES

## Abstract

There is an increasing need to use genome and transcriptome sequencing to genetically diagnose patients suffering from suspected monogenic rare diseases. The proper detection of compound heterozygous variant combinations as disease-causing candidates is a challenge in diagnostic workflows as haplotype information is lost by currently used next-generation sequencing technologies. Consequently, computational tools are required to phase, or resolve the haplotype of, the high number of heterozygous variants in the exome or genome of each patient. Here we present SmartPhase, a phasing tool designed to efficiently reduce the set of potential compound heterozygous variant pairs in genetic diagnoses pipelines. The phasing algorithm of SmartPhase creates haplotypes using both parental genotype information and reads generated by DNA or RNA sequencing and is thus well suited to resolve the phase of rare variants. To inform the user about the reliability of a phasing prediction, it computes a confidence score which is essential to select error-free predictions. It incorporates existing haplotype information and applies logical rules to determine variants that can be excluded as causing a recessive, monogenic disease. SmartPhase can phase either all possible variant pairs in predefined genetic loci or preselected variant pairs of interest, thus keeping the focus on clinically relevant results. We compared SmartPhase to WhatsHap, one of the leading comparable phasing tools, using simulated data and a real clinical cohort of 921 patients. On both data sets, SmartPhase generated error-free predictions using our derived confidence score threshold. It outperformed WhatsHap with regard to the percentage of resolved pairs when parental genotype information is available. On the cohort data, SmartPhase enabled on average the exclusion of approximately 22% of the input variant pairs in each singleton patient and 44% in each trio patient. SmartPhase is implemented as an open-source Java tool and freely available at http://ibis.helmholtz-muenchen.de/smartphase/.

This is a *PLOS Computational Biology* Software paper.

## Introduction

Genetic defects are the source of a wide and diverse range of monogenic or Mendelian diseases that are individually rare but collectively common. So far, more than 5, 000 different disorders and traits are known that are caused by mutations in only one gene [1]. Genome as well as transcriptome sequencing is increasingly used to genetically diagnose patients suffering from a suspected monogenic rare disease [2–4]. However, detecting disease-causing variants among thousands of benign variants is a great challenge. Widely-used strategies and guidelines for variant prioritization are based on the predicted or known deleteriousness of a variant, its frequency in large scale sequencing studies and its segregation with the disease phenotype [5, 6]. Assuming autosomal recessive monogenic inheritance, the disease-causing variants are either homozygous or compound heterozygous with two heterozygous mutations together affecting both parental alleles of a gene locus [7]. Consequently, clinical workflows aim to detect with preference rare variants that are predicted to be harmful and are homozygous or compound heterozygous in the patient. The accurate determination of whether two heterozygous variants are located on the same or different parental alleles is a challenge that is faced by all diagnostic pipelines in the context of recessive monogenic diseases.

Haplotypes can either be resolved experimentally during sequencing or inferred computationally afterwards [8]. Several technologies for haplotype-resolved genome sequencing have been developed but are seldom used in a clinical setting because of their prohibitive cost and complexity. Computational tools for phasing use sequencing data of family members, reads spanning multiple variants or reference haplotype panels. Sequencing data of parents or other family members is most informative for phasing but might not always be available and cannot be used for variants that are heterozygous in both parents and the child. Using reads spanning multiple variants requires no additional data, but the length of the underlying reads limits the number of variants that can be phased. Panel-based phasing methods are useful for common variants but fail for rare variants which are the focus when diagnosing rare diseases. The combination of different phasing strategies is promising as it can compensate for the disadvantages of the individual approaches.

Existing phasing tools offer limited utility for clinical purposes because they are designed to phase complete chromosomes instead of genetic loci of interest or incorporate only one phasing strategy. phASER improves the phasing range of read-based phasing by incorporating RNA sequencing reads in addition to DNA sequencing reads, but it does not perform pedigree-based phasing [9]. WhatsHap combines read-based phasing with pedigree-based phasing but offers no options to restrict phasing to pre-selected variants or genomic regions [10]. The user would either have to accept unnecessarily long runtimes for phasing complete chromosomes or trim the sequencing data to the regions of interest before each execution which would require additional time and storage resources. Neither option is feasible in a clinical setting especially when dealing with large cohorts of thousands of patients with few regions of interest. Furthermore, none of these phasing tools are able to label pairs of heterozygous variants as clinically irrelevant by using the fact that the genotypes of healthy parents contradict the potential pathogenicity of the pair.

To overcome these limitations, we developed SmartPhase, a ready-to-use phasing tool tailored for clinical workflows to improve the analysis of potential compound heterozygous variant pairs in terms of simplicity, speed and accuracy. SmartPhase is able to flexibly use available trio sequencing information and read information of DNA as well as RNA sequencing data. Additionally, it informs about the confidence of its predictions and implements rules to logically exclude variant constellations that cannot be disease-causing.

## Design and implementation

To fully take advantage of the breadth of phasing informative data generated in clinical research, SmartPhase is able to combine trio phasing, read-based phasing, additional logical rules and GATK physical phasing to resolve as many variant combinations as non-pathogenic as possible. Furthermore, SmartPhase focuses on diagnostically relevant genomic loci in its input while providing a comprehensive bitflag and confidence score system to intuitively represent its results in its output.

### Trio phasing

If parental genotypes are provided, all patient heterozygous variants are examined for the possibility of using the parental variant calls to allocate a variant to either the maternal or paternal chromosome. If the pedigree information allows the phase to be determined, a confidence score of 1.0 is given.

Generally, it is not possible to assign *de novo* mutations to the correct allele by trio information. However, if the inherited allele is present in both parents, with one being heterozygous and the other being homozygous for said allele, it is probabilistically assigned to the homozygous parent with a confidence score of 0.66.

### Read-based phasing

If DNA or RNA sequencing reads are provided, SmartPhase uses multi-variant spanning reads to aid in the resolution of local haplotypes. If a read spans two variant positions and contains both variants, this constitutes an evidence that both variants lie on the same allele in *cis* configuration. If two reads span both variant positions, each containing only one of the variants, this is an evidence that the variants lie on opposing chromosomes in *trans* configuration. To ensure the creation of accurate haplotypes, SmartPhase ignores reads that are not mapped, not part of a proper pair in case of paired-end reads, marked as duplicate reads, or not part of a primary alignment. Further, the user can choose to ignore reads whose mapping quality is lower than that of a defined threshold.

In order to inform about the quality of the inferred haplotype for two variants *v*_1_ and *v*_2_, a confidence score, Confidence(*v*_1_, *v*_2_), is computed by the formula

$$\frac{\left|\overset{\text{Trans}\;\text{Subscore}}{\overset{︷}{\sum_{i=1}^{n_{trans}}\left(1-\underset{\text{Average}\;\text{Inverse}\;\text{Phred}}{\underset{︸}{\frac{\overset{\text{Corrected}\;v_{1}}{\overset{︷}{\frac{\sum_{k=1}^{l_{v_{1}}}10^{-q_{k}/10}}{l_{v_{1}}}}}+\overset{\text{Corrected}\;v_{2}}{\overset{︷}{\frac{\sum_{k=1}^{l_{v_{2}}}10^{-q_{k}/10}}{l_{v_{2}}}}}}{2}}}\right)}}-\overset{\text{Cis}\;\text{Subscore}}{\overset{︷}{\text{min}(2\sum_{j=1}^{n_{cis}}(1-\underset{\text{Average}\;\text{Inverse}\;\text{Phred}}{\underset{︸}{\frac{\overset{\text{Corrected}\;v_{1}}{\overset{︷}{\frac{\sum_{k=1}^{l_{v_{1}}}10^{-q_{k}/10}}{l_{v_{1}}}}}+\overset{\text{Corrected}\;v_{2}}{\overset{︷}{\frac{\sum_{k=1}^{l_{v_{2}}}10^{-q_{k}/10}}{l_{v_{2}}}}}}{2}}}),n)}}\right|}{n+2}$$ (1)

where *n* is the number of reads overlapping two variant positions, *n*_*trans*_ is the number of reads supporting a *trans* configuration where each variant is present in only half of the reads, *n*_*cis*_ is the number of reads containing both variants, *q*_*k*_ is the Phred quality score of a read at a particular position *k*, and *l* represents the length of the variant allele being examined in this read (either *v*_1_ or *v*_2_). The confidence score was designed to summarize the strength of the evidence behind a read-based phasing call. For a thorough explanation of how the confidence score is calculated, see Section 1.1 of S1 Appendix. In order to differentiate high quality phasing calls from low quality phasing calls, we derived 0.34 as a threshold for the confidence score as explained in Section 1.2 of S1 Appendix.

Variants are directly phased with their immediate neighbor using the *cis* and *trans* subscores, calculated by summing the inverse Phred score corrected evidence counts. This basic strategy permits the creation of seed haplotypes that can be locally extended to neighboring variants if overlapping reads exist. SmartPhase can elongate these haplotypes by applying this strategy within paired-end reads as the two paired-end reads must come from the same haplotype, regardless of physical distance or disjointedness. SmartPhase also leverages RNA sequencing reads that connect distant variants due to the read spanning exon-exon boundaries. To calculate the confidence of non-directly phased variants, the confidence scores of all directly phased variants on the shortest path are multiplied together. This helps to represent the growing uncertainty of phase calls as haplotype blocks increase in size and the distance between variants increases.

### Phasing intervals

If both reads as well as parental variants are provided, the local haplotype blocks created by read-based phasing are combined using variants that were pedigree phased. Any contradictions between pedigree phasing and read-based phasing are resolved according to their confidence scores. All variant pairs not phased by direct evidences again have their confidence scores calculated by taking the product of all directly phased linking variants on the shortest possible path between the variants in the pair.

### Innocuous labeling

If parental genotype information is given, certain variant pair constellations can be designated as *innocuous* based on the assumption that the parents of the patient are healthy. *Innocuous* variants are those variants that are deemed to be clinically irrelevant as all variant combinations they partake in have been deduced to be non-disease-causing in Section 1.3 of S1 Appendix. Variant pairs are labeled as *innocuous* if one of the variants is homozygous in a parent or if mother, father, and child all possess the same heterozygous genotype for one of the variants in the pair.

### GATK physical phasing

If a variant is not visible in the alignment of the reads as given by the provided mapping file, this variant was most likely called as a result of read realignment done by the used variant calling program. As a consequence, these variants are designated as not found by SmartPhase. As the HaplotypeCaller (HC) tool of the Genome Analysis Toolkit (GATK) [11] is currently one of the most widely-used variant calling algorithms, we implemented the ability to incorporate phasing information returned by HC when no variant evidences were found within the reads and the variant could not be phased by trio phasing. If HC calls variants through read rearrangement, these variants are usually physically phased at the same time and the used local haplotype information is provided in the resulting variant file. The phase of otherwise missing variants is adopted from the variant files and given a confidence score of 1.0.

### Input & output

SmartPhase resolves haplotypes in genomic intervals of interest. Genomic intervals can either be directly defined by the user, or are generated by creating regions enveloping potential compound heterozygous variant pairs of interest. SmartPhase accepts up to two variant specifying files encompassing all variants and those that have been filtered to be deemed clinically relevant. The all variants file is used to create haplotype blocks and usually corresponds to the result of variant calling. The filtered variants file is optional, but can be used to narrow the scope of the output as only those variants specified in this file are printed in the final result. These variants generally constitute the set of variants that were filtered for clinical relevance according to allele frequency, predicted functional impact and other criteria. As many mapping files containing DNA or RNA sequencing reads as desired can be provided to be used during read-based phasing.

**Fig 1:**
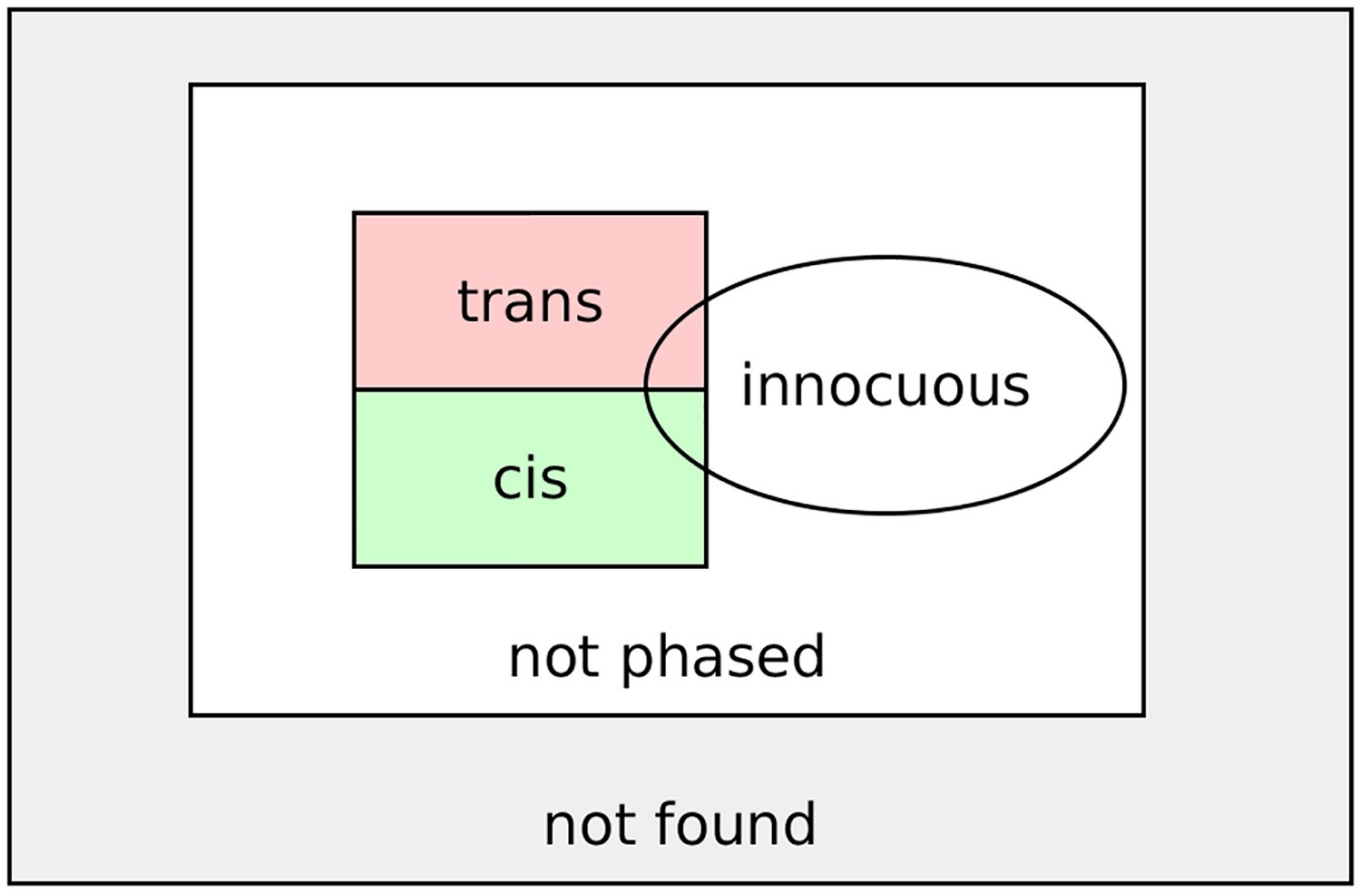
Visualization of the bit flag system. If a variant pair could be phased, it is either labeled as *cis* or *trans*. Additionally, it can be labeled as *innocuous*. If a variant pair could not be phased, there was either too little evidence for calling *cis* or *trans* or one of both variant alleles could not be found in the mapped reads.

The phases and confidence scores of all heterozygous variant combinations within the given or created intervals are the results of SmartPhase. To fully capture the complexity of phased variant pairs, *innocuous* pairs, and variants that were not found in the mapping data, we developed a bitwise flag system to efficiently store all necessary information in a single number. The classification of variant pairs according to the defined criteria is visualized in Fig 1. Table A of S1 Appendix shows the possible combinations of bits and the corresponding final flag.

## Results

We validated SmartPhase on simulated as well as real clinical whole-exome sequencing (WES) data to show its accuracy and compare its performance to WhatsHap. We benchmarked both tools using the runtime, the number of *innocuous* and phased pairs, and the proportion of incorrect predictions. As variant pairs phased or labeled as *innocuous* are equally informative for diagnostic workflows, we summarize these by the term “*cleared* pairs”. We refer to them as *confidently cleared* pairs after removing low quality calls with a confidence score below 0.34 as derived in Section 1.2 of S1 Appendix. In the context of clinical diagnosis, we are especially interested in variant pairs that can be excluded as being non-disease-causing. Variant pairs can be designated as being clinically non-relevant by *innocuous* labeling or by being on the same allele through confident *cis* calls which we sum up as *non-pathogenic* pairs.

### Comparison of SmartPhase to WhatsHap on simulated data

As described in Section 2 of S1 Appendix, we simulated WES data of the widely used CEU and YRI trio and phased their heterozygous variants in genes on chromosome 1 and 19 using SmartPhase and WhatsHap. We generated a set of 26, 638 potential heterozygous variant pairs distributed over 2, 922 genes with 4.21 heterozygous variants per gene on average (see Table B of S1 Appendix). Table 1 shows an overview of the main results of the benchmark (complete data in S1 Table).

**Table 1:** Benchmark results of SmartPhase and WhatsHap.

Scenario				SmartPhase						WhatsHap			
Trio	Chr	Pairs	Mode	*Confidently cleared* in %	*Innocuous*	Phased	Err	LQ	Time in S	Phased in %	Phased	Err	Time in S
CEU	1	6, 783	*read only*	12.1	0	953	14	130	49.3	14.9	1, 010	5	274.3
CEU	19	4, 531	*read only*	17.2	0	891	4	110	31.0	20.8	940	2	157.6
YRI	1	9, 186	*read only*	14.3	0	1, 462	4	146	62.4	17.4	1, 595	7	288.0
YRI	19	6, 138	*read only*	17.2	0	1, 198	3	141	37.2	20.3	1, 248	3	169.6
CEU	1	6, 783	*read & trio*	100.0	4, 700	2, 083	0	0	51.3	79.6	5, 399	74	270.2
CEU	19	4, 531	*read & trio*	100.0	3, 087	1, 444	0	0	31.1	85.2	3, 860	66	159.7
YRI	1	9, 186	*read & trio*	100.0	5, 618	3, 568	0	0	63.0	88.0	8, 085	153	282.7
YRI	19	6, 138	*read & trio*	100.0	3, 739	2, 399	0	0	38.3	89.1	5, 470	101	168.1

We compared SmartPhase (SP) to WhatsHap (WH) using the number of phased pairs, the number of incorrect phased pairs (Err), and the runtime on the same processing node, measured in seconds, as benchmark parameters. Variant pairs that were both labeled as *innocuous* and phased were only counted as *innocuous*. Pairs that were phased with a confidence score below 0.34 are counted as low quality (LQ) pairs.

Phasing in *read only* mode results in low amounts of phased pairs for both phasing tools. In comparison to WhatsHap, SmartPhase clears 2.8%–3.6% less pairs because variant alleles are often not found in the mapped reads as reported in the variant file. Variant calling tools, like the GATK HaplotypeCaller, rearrange read alignments internally before calling variants. As SmartPhase does not realign reads in contrast to WhatsHap, it performs worse in areas of uncertain mapping when only read information is provided. The results for phasing based on read and trio information demonstrate the power of SmartPhase, as the combination of *innocuous* labeling and phasing clears all input variant pairs. This corresponds to 10.9%–20.4% more variant pairs cleared in comparison to WhatsHap.

SmartPhase generated error-free predictions in the combined *read & trio* mode and all errors in *read only* mode are labeled as low quality. WhatsHap has an average error-rate of 1.03% (0.21%–1.89%) with a remarkable increase of the error rate in combined *read & trio* mode. Although this number is quite low, it can have detrimental consequences if even only one pair is wrongly predicted as being in *cis* configuration when in reality it is disease-causing. This emphasizes how crucial a confidence score is in generating trustworthy and accurate predictions.

Another advantage of SmartPhase is its runtime which is on average five times faster than WhatsHap, independent of using only read or both read and trio information. While the absolute difference is minor for the limited, simulated data, the runtime becomes particularly relevant in a clinical setting where variant pairs from all chromosomes of hundreds of patients must be phased.

### Validation of SmartPhase on clinical WES data

We validated SmartPhase on a cohort of clinical WES data that consists of 121 trio and 800 singleton patients without parental genotype information. As detailed in Section 3 of S1 Appendix, we selected a set of 116, 613 potential compound heterozygous variant pairs after filtering for rare and protein-altering heterozygous autosomal variants. On average, we identified 126.62 ± 161.25 variant pairs per individual.

#### Overall performance of SmartPhase

**Fig 2:**
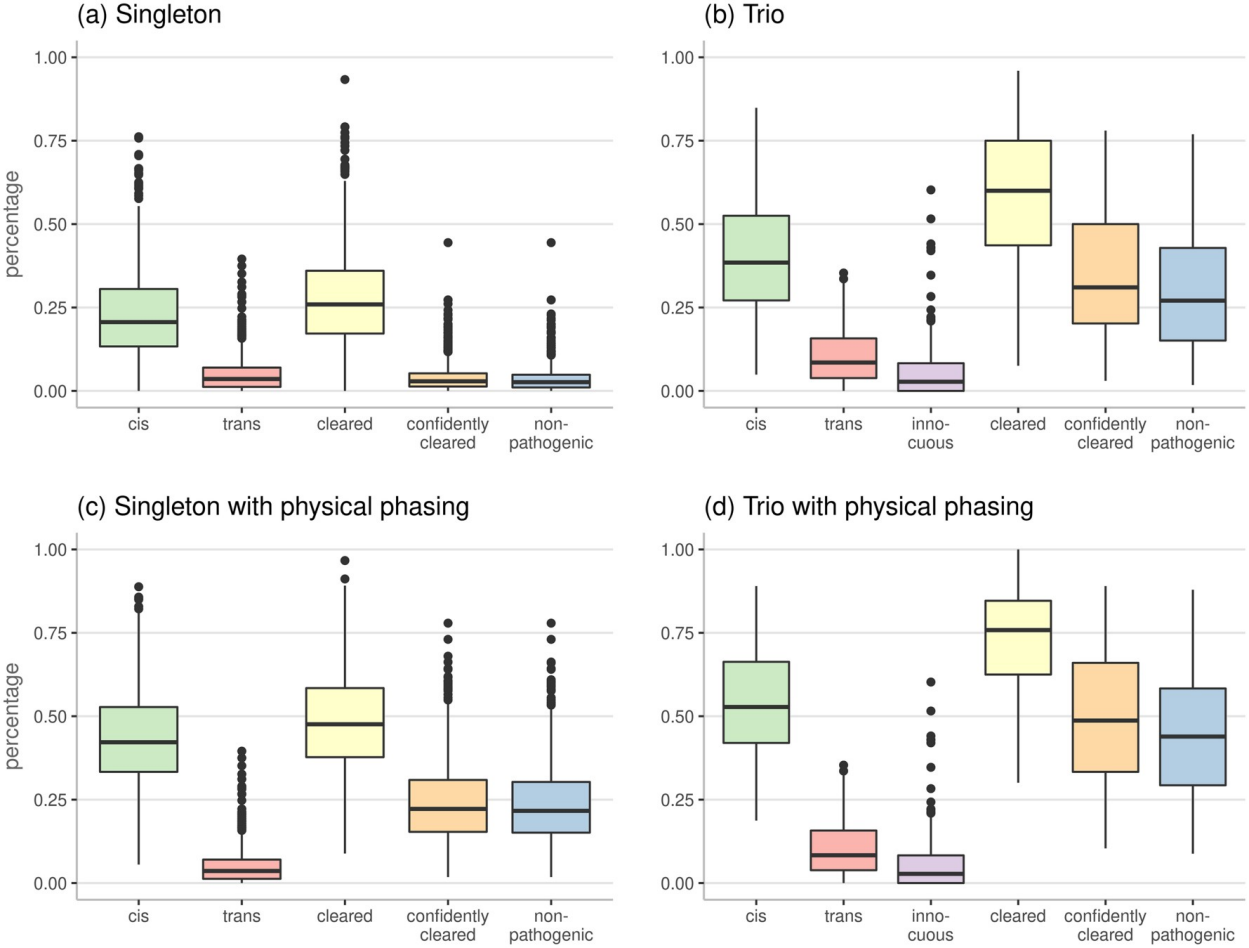
Boxplots showing the distribution of relative amounts of pairs labeled as *cis*, *trans*, and *innocuous* (only for trio phasing) as well as the percentages of pairs that are *cleared*, *confidently cleared* after removing low quality phasing predictions, and pairs that can be excluded as being *non-pathogenic*. The plots show results for SmartPhase using only read information for 800 singleton patients (a), using both trio and read phasing for 121 trio patients (b) and the results for the same individuals using physical phasing information provided by the HaplotypeCaller of GATK (c) & (d).

To evaluate the overall performance of SmartPhase on real data, we applied it to all 116, 613 variant pairs identified in the 921 individuals of the cohort with a runtime of 190 minutes for all patients or 12 seconds per patient. The results with and without physical phasing for both singleton and trio patients are shown in Fig 2 (complete data in S2 Table).

For singleton patients, in median 25.91% of all variant pairs can be cleared by being phased (see Fig 2(a)). Ignoring low quality phase predictions with a confidence score below 0.34 results in 2.88% phased pairs. The median fraction of *non-pathogenic* variants is slightly lower at 2.62%.

For trio patients, in median 60.00% of all input pairs are cleared (see Fig 2(b)). The percentage is lower than in the simulated data where all variant pairs could be cleared. As real WES data is imperfect due to failures in exome capturing or low coverage, some genotypes are missing in one or both of the parents which may make a variant pair impossible to resolve. In our simulated data, the largest fraction of pairs is cleared due to *innocuous* labeling. In our clinical data, it corresponds to the smallest fraction of *cleared* pairs. This is due to the preceding filtering for rare variants which are unlikely to be homozygous in one parent or heterozygous in both parents (see Section 3.2 of S1 Appendix). Removing low quality phase predictions results in 31.03% of the pairs being cleared. In median, 27.06% of all input variant pairs are *non-pathogenic*.

To enhance the power of SmartPhase it is able to incorporate phasing information generated by the HaplotypeCaller of GATK. For both phasing of singleton and trio patients, Fig 2(c) and 2(d) show a noticeable increase in *cis* calls indicating that GATK physical phasing mostly informs about variants being on the same allele, as expected. The percentage of pairs that can be considered clinically irrelevant increases from 2.62% to 21.64% for singletons and from 27.06% to 43.91% for trio patients.

#### Comparison of SmartPhase to WhatsHap

**Fig 3:**
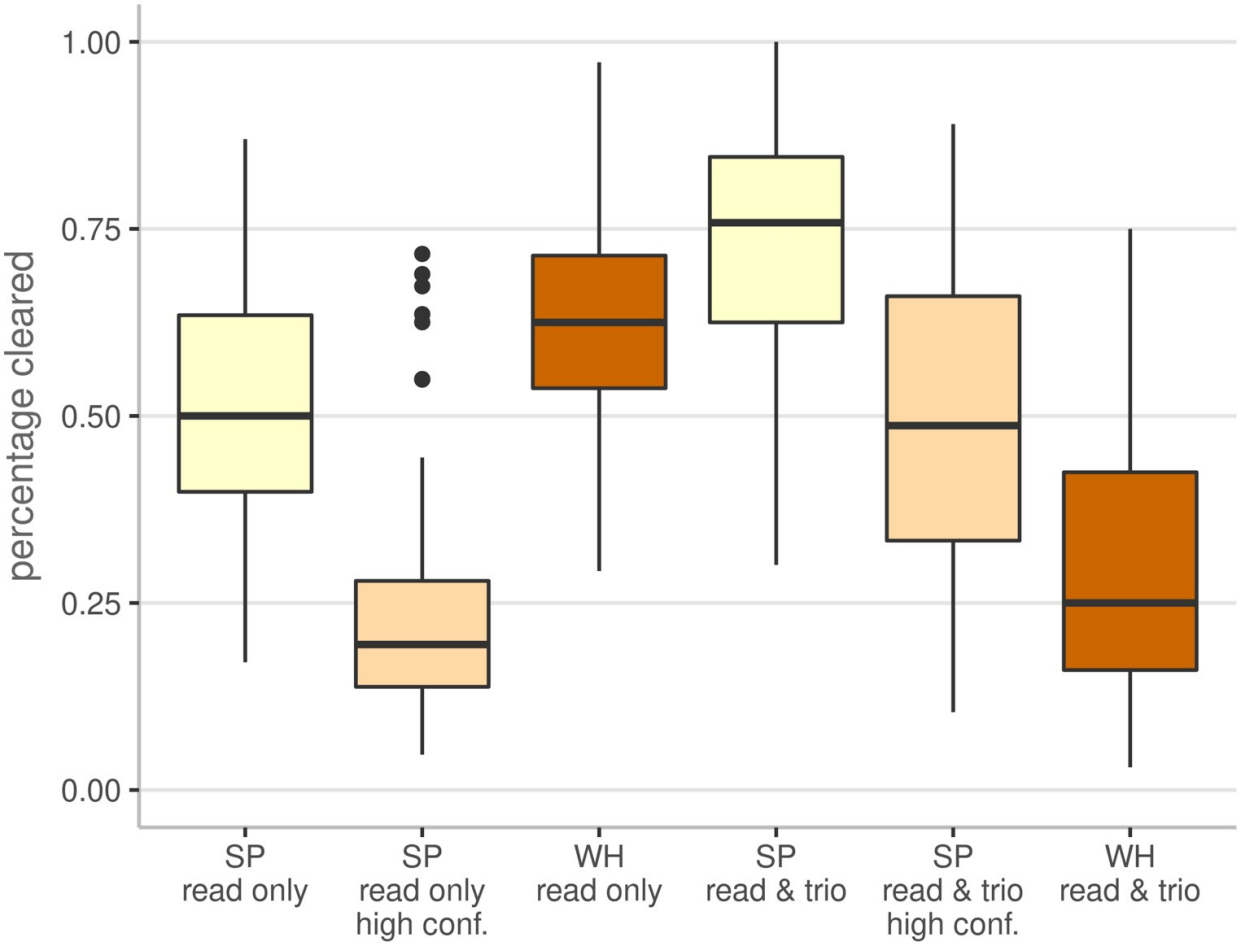
Boxplots showing the percentage of *cleared* pairs for SmartPhase (SP) and WhatsHap (WH) in *read only* and in combined *read & trio* mode on the 21, 066 variant pairs identified in the 121 trio patients of the clinical WES data cohort.

In order to extend the comparison of SmartPhase and WhatsHap to real data, we applied both tools in *read only* and in combined *read & trio* mode to 21, 066 variant pairs in 121 trio patients of the clinical WES cohort. On the reduced data set, WhatsHap required 90 hours to phase the patients making an analysis of the entire cohort of 921 patients not realistic as it would take approximately 585 hours or more than 28 days. Reducing the input files to the regions of interest would shorten the runtime but requires additional preprocessing steps consuming time and storage resources also resulting in an unrealistic scenario. Fig 3 shows the distribution of the percentages of *cleared* pairs for each trio patient for both tools in both modes (complete data in S3 Table).

The performance in *read only* mode is in the same range for SmartPhase and WhatsHap with 50.0% and 62.5% in median. When removing low confidence predictions of SmartPhase with a confidence score below 0.34 the percentage of *cleared* pairs decreases to a median of 19.4% with some outliers still showing a similar performance to WhatsHap.

Examining a subset of 4, 701 variant pairs that can be phased using parental genotype information, SmartPhase generated phase predictions for 1, 577 pairs with 246 pairs being phased confidently. All of these confident phasing predictions are consistent with parental genotypes. For the 1, 331 pairs with a confidence score below 0.34, only 63 predictions are inconsistent with parental genotypes. For 1, 491 pairs, the aligned reads do not contain at least one of both variants. WhatsHap in comparison generated phase predictions for 3, 058 pairs with 34 erroneous calls. Even though WhatsHap phased markedly more variant pairs, it is not possible to filter out incorrect calls. This can have serious clinical effects, especially when considering that 15 of the 34 errors are *cis* calls that are in fact compound heterozygous according to the parental genotypes.

In combined *read & trio* mode SmartPhase increased the percentage of *cleared* variant pairs considerably to a median value of 75.8% before and 48.7% after confidence score filtering. WhatsHap performed remarkably worse when parental genotype data is provided. The phasing rate drops from 62.5% to 25.0% because WhatsHap ignores variants when genotypes are missing in the parents or contradicting Mendelian inheritance is observed.

#### Summary

The validation on simulated and clinical data confirms that SmartPhase is characterized by a fast and highly accurate performance. We demonstrated that using a confidence score threshold of 0.34 generates error-free predictions for the complete simulated data set and a sub-set of the clinical data. We showed that SmartPhase outperforms WhatsHap when both read and trio information is provided due to *innocuous* labeling and WhatsHap’s strict handling of variants with missing genotypes in the parents or those violating Mendelian rules. If only read-based phasing is possible, SmartPhase clears less pairs than WhatsHap as SmartPhase takes the provided read alignments as is without performing any realignment. However, as SmartPhase compensates by incorporating phasing information generated by GATK HC, it can approximate WhatsHap’s read-based phasing performance. As GATK HC is the currently prevailing variant caller, physical phasing information of realigned reads can be exploited in most pipelines. Not performing computationally expensive read realignment allows SmartPhase to be markedly faster which is crucial when considering rapidly growing clinical patient cohorts. As the average read length of sequencing techniques is constantly increasing, read mapping will become more and more accurate without the need for realignment, putting SmartPhase at a clear advantage.

As a stand-alone Java application, SmartPhase can be seamlessly incorporated into any clinical workflow without requiring further installations or downloads. Taken together, SmartPhase greatly simplifies the selection of potential compound heterozygous variant pairs as disease candidates and reduces the search space for pathogenic compound heterozygous variant pairs considerably. The resulting speed up of the analysis of clinical sequence data is helpful for all patients, even if their disease is not caused by a compound heterozygous variant.

## Availability and future directions

The source code of SmartPhase, its documentation, a minimum test data set, and the complete validation pipeline that generates and evaluates simulated data are provided in S1 Code and S1 Text. Additionally, all files together with the simulated data can be found at http://ibis.helmholtz-muenchen.de/smartphase/. Comprehensive results of SmartPhase using simulated data and for each of the 921 individuals of the cohort are available in S1, S2 and S3 Tables.

Besides the demonstrated use of SmartPhase in detecting and filtering compound heterozygous variants, it can further be used for the analysis of multi-nucleotide variants that have been shown to play an important role in human diseases [12]. As the detection of multi-nucleotide variants requires phasing of nearby variants, SmartPhase is perfectly suited for this clinical task. SmartPhase was designed to be easy to use in any existing clinical sequencing data workflow. To increase the usability even more, we plan to make SmartPhase available as a module for analysis platforms like Galaxy [13] or KNIME4NGS [14]. Beyond that, the phasing efficacy of SmartPhase can be improved by the integration of panel-based phasing methods enabling the connection of haplotype blocks by phasing of common variants.

### Ethics statement

The underlying studies that generated human exome sequencing data that were used for the manuscript were approved by the Ethics Commission of the Medical Faculty of the LMU Munich. The reference numbers of the ethics votes are 346-11, 381-11, 387-11, 438-11, 486-11, 303-12, 187-13 BB, 353-13, 66-14, 501-14 and 806-16. The consent was obtained in written form.

## Supporting information

S1 Appendix Additional information on methodology and validation. The first part provides details on the confidence score formula, the derivation of the confidence score threshold, *innocuous* labeling and the list of all possible bitflags generated by SmartPhase. The second part describes the generation of simulated WES data, the configuration of SmartPhase and WhatsHap, the selection of candidate variant pairs. The third part describes the processing of clinical WES data, the filtering of candidate variant pairs and gives general information on the performed validations.
(PDF)

S1 Table Results of SmartPhase and WhatsHap on simulated data. Results of our benchmark of SmartPhase on simulated data.
(XLSX)

S2 Table Results of SmartPhase on clinical WES data. Results of our application of SmartPhase to the cohort of clinical WES data.
(XLSX)

S3 Table Results of SmartPhase and WhatsHap on trio patients. Results of our comparison of SmartPhase to WhatsHap on trio patients of the clinical WES data cohort.
(XLSX)

S1 Code Source code files of SmartPhase. In addition to the source code, the archive file contains the scripts of the validation pipeline and a minimum test data set.
(ZIP)

S1 Text Documentation of SmartPhase. The documentation gives instructions on how to run SmartPhase and how to interpret its results.
(PDF)
