# Supplementary material for: SmartPhase: Accurate and fast phasing of heterozygous variant pairs for genetic diagnosis of rare diseases
Source: PLoS Comput Biol. 2020 Feb 7;16(2):e1007613. doi: 10.1371/journal.pcbi.1007613 (PMC7032729; doi:10.1371/journal.pcbi.1007613)
Supplement: S1 Appendix — The first part provides details on the confidence score formula, the derivation of the confidence score threshold, innocuous labeling and the list of all possible bitflags generated by SmartPhase. The second part describes the generation of simulated WES data, the configuration of SmartPhase and WhatsHap, the selection of candidate variant pairs. The third part describes the processing of clinical WES data, the filtering of candidate variant pairs and gives general information on the performed validations. (PDF) [file pcbi.1007613.s001.pdf]

Appendix SmartPhase

Paul Hager, Hans-Werner Mewes, Meino Rohlfs, Christoph Klein, Tim Jeske

January 29, 2020

1 Design and Implementation

In the subsequent sections we describe in detail the reasoning behind the confidence score formula, the confidence score threshold of 0.34 and the innocuous labeling feature of SmartPhase. Further, a tabular overview of all possible outcomes of the implemented bitflag system is given.

1.1 Confidence score for read-based phasing

A confidence score formula was developed to help the user make informed decisions about which phasing calls can be trusted and which should be cautiously examined before further analysis.

The formula for Confidence(v_1, v_2) is

$$\frac{\left| \overbrace{\sum_{i=1}^{n_{trans}} (1 - \underbrace{\frac{\overbrace{\frac{\sum_{k=1}^{l_{v_1}} 10^{-q_k/10}}{l_{v_1}}}^{\text{Corrected } v_1} + \overbrace{\frac{\sum_{k=1}^{l_{v_2}} 10^{-q_k/10}}{l_{v_2}}}^{\text{Corrected } v_2}}{2}}_{\text{Average Inverse Phred}})}^{\text{Trans Subscore}} - \overbrace{\min(2\sum_{j=1}^{n_{cis}} (1 - \underbrace{\frac{\overbrace{\frac{\sum_{k=1}^{l_{v_1}} 10^{-q_k/10}}{l_{v_1}}}^{\text{Corrected } v_1} + \overbrace{\frac{\sum_{k=1}^{l_{v_2}} 10^{-q_k/10}}{l_{v_2}}}^{\text{Corrected } v_2}}{2}}_{\text{Average Inverse Phred}}), n)}^{\text{Cis Subscore}} \right|}{n+2} \tag{1}$$

where n is the number of reads overlapping two variant positions, n_{trans} is the number of reads supporting a *trans* configuration while giving equal evidence to both variants, n_{cis} is the number of reads containing both variants, q_k is the Phred quality score of a read at a particular position k, and l represents the length of the variant allele being examined in this read (either v_1 or v_2).

The Phred quality score assesses the quality of base determination during sequencing and is logarithmically related to the base-calling error probability. A Phred quality score of 10 corresponds to a base call accuracy of 90% while a Phred quality score of 60 represents a base call

accuracy of 99.9999%. The inverse of the Phred quality score, the base-calling error probability, is used to weaken the evidences of reads with poor base qualities. It is averaged over all positions in the first variant in a read to deliver the corrected v_1 score which is then averaged with the corrected v_2 score to give the average inverse Phred score. This is then subtracted from one to give the Phred adjusted evidence count. For single nucleotide polymorphisms and deletions, l_v will be 1, but for insertions it is important to examine all positions, which is why the variant is corrected over its length. The Phred adjusted evidence counts are summed over all reads which cover this variant and support the same *cis* or *trans* call, giving the *cis* and *trans* subscores. Thus, the higher the average Phred score per read, the closer the *trans* and cis subscore sums are to n_{trans} and n_{cis}. This can be seen when considering a maximal Phred score of 60, which will result in an average inverse Phred score of $1\text{x}10^{-6}$, which will minimally lower the *trans* or *cis* subscores away from n_{trans} or n_{cis}. This penalizes variants with poor call quality and dramatically reduces the chance of equal evidences for *cis* and *trans* being seen, which could be the case if simply n_{trans} and n_{cis} are used.

In the case of compound heterozygosity, it is expected that half of all reads contain only the first variant while the other half contain only the second variant. To reflect this in the score and to prevent *trans* evidences of only one of both variants being considered equal to evenly distributed evidences of each variant, the lists of variant evidences are sorted by corrected score and the longer list is reduced to one larger than the size of the smaller list by removing those variants with lowest scores.

In the case of two variants being *cis*, it is expected that half of all reads will contain both variant alleles, and the other half will contain both reference alleles. As reads containing both reference alleles constitute only ambiguous evidences for real germline mutations, only those containing both variants are counted. When substantially more than half of all reads contain both reference alleles, this may be either an indication of a mosaic and not a germline mutation or simply weak evidences for variants at these positions. Both of these are guarded against by counting only those evidences which exhibit both variants. This results in approximately half of all reads increasing the Phred adjusted evidence counts for a pair of variants that lie on the same chromosome under normal conditions. The minimum between twice the Phred adjusted evidence counts and n is taken to prevent a confidence greater than one being given, if more than half of all reads contain both variants.

The denominator is increased by two to prevent a small amount of reads from being able to achieve a high confidence score, thus asymptotically increasing the confidence towards 1.0 as the amount of reads increases without any conflicting evidence towards the supported phase. Specifically two was chosen to prevent a single false evidence of giving a score close to 0.5, rather pushing it towards 0.33. The more contradicting evidences found for both *cis* and *trans* configurations of two variants, the lower the confidence score.

1.2 Confidence score threshold for reliable phasing calls

For variant pairs for which there are either only *cis* or only *trans* evidences, the confidence score indicates the fraction of the reads that are counted as evidence. For variant pairs for which there

are both *cis* and *trans* evidences, the confidence score is determined by the absolute difference of the conflicting evidences. Besides the difference of evidences, the ratio of *cis* to *trans* evidences needs to be taken into account to define confident phasing calls. In order to determine a confidence score threshold, we analyze the behaviour of the confidence score in the presence of conflicting evidences. To simplify the subsequent calculations we use a simplified version of equation 1

$$\frac{|n_t - n_c|}{n_t + n_c + 1} \tag{2}$$

where we assume that only direct evidences for either a *cis* or *trans* configuration exist and thus n_t is the number of reads supporting a *trans* configuration and n_c is the sum of the reads containing both variants or no variant. In comparison to equation 1, there is no Phred quality score correction which corresponds to a situation of perfect quality reads.

We define a phase call as reliable when there are at least twice as many evidences for one variant constellation in comparison to the other. Assuming, without loss of generality, that there are exactly twice as many evidences for a *trans* constellation, $n_t = 2n_c$, equation 2 simplifies to

$$\frac{n_c}{3n_c + 1} \tag{3}$$

with n_c ranging from 1 to infinity. Consequently, the confidence score starts at $\frac{1}{4}$ for $n_c = 1$ and approaches $\frac{1}{3}$ for infinitely large n_c. As we want to identify a threshold for the confidence score, we are searching for the maximum confidence score that can be obtained while keeping the ratio of evidences slightly below 2, corresponding to a phasing call that we want to consider as a low quality call. Such ratios can easily be generated by adding 1 to the *cis* evidences which changes equation 3 to

$$\frac{n_c - 2}{3n_c - 1} \tag{4}$$

with n_c ranging from 2 to infinity. Consequently, the confidence score starts at 0 for $n_c = 2$ and approaches $\frac{1}{3}$ when the ratio of *trans* and *cis* evidences is below 2. Conversely, this means that a confidence score larger than $\frac{1}{3}$ can only be achieved when there are more than twice as many evidences for a configuration. This can easily be shown by generating ratios slightly larger than 2 by adding 1 to the *trans* evidences which changes equation 3 to

$$\frac{n_c + 1}{3n_c + 2} \tag{5}$$

with n_c ranging from 1 to infinity. Now, the confidence score starts at 0.4 for $n_c = 1$ and approaches $\frac{1}{3}$ while being always larger than $\frac{1}{3}$.

As a result, we recommend a minimum confidence score of 0.34 for assessing the quality of phasing calls of SmartPhase. This ensures that there are more than twice as many evidences for one constellation in the case of conflicting evidences. Furthermore, this ensures that at least $\frac{1}{3}$ of the reads are counted as evidences in the case of evidences for either only a *cis* or a *trans* constellation.

1.3 Innocuous labeling

The primary weakness of trio phasing is its inability to phase when mother, father, and child all possess the same heterozygous genotype. An advantage of SmartPhase is that it operates within a defined task, namely determining the relevance of heterozygous variants with regards to disease-causing compound heterozygosity. This allows SmartPhase to use logically derived rules to further reduce the pool of disease-causing variant combinations. If trio information is provided, through careful analysis of the parent genotypes and possible inheritance patterns, a large portion of previously unphasable variants can be labeled as *innocuous*.

For the following, six primary assumptions were made:

Assumption 1. *No meiotic recombination events have taken place within the gene.*

Assumption 2. *The effect of two equally deleterious compound heterozygous variants is the same as either being homozygous.*

Assumption 3. *The genotype information is as called.*

Assumption 4. *The parents do not possess the pathogenic phenotype of the patient.*

Assumption 5. *The probabilities and effects of inheritance of one parent are equal to those of the other parent.*

Assumption 6. *Within a parent or patient, the probabilities and effects of inheritance of one chromosome are equal to those of the other chromosome.*

In support of assumption 1, it has been shown that in humans meiotic recombination primarily occurs at intergenic CCN-like motifs [1]. In addition, it has been observed that meiotic recombination hotspots are preferentially located outside the transcribed domain [2].

Assumption 2 is based on the findings that in the case of Mendelian human disease as well as human diseases such as Autism spectrum disorder, compound loss-of-function heterozygosity is equally as harmful as a homozygous loss-of-function variant [3] [4] [5].

Assumption 3 is taken for the sake of simplicity and because stringent filtering has been done to reduce the presence of false positives. In addition, marking false positive variants for filtration is still of assistance in lightening the amount of downstream analysis required without losing actual possible disease-causing variants.

Assumption 4 stems from the fact that compound heterozygous variants are primarily of interest in cases when the parents do not show the negative phenotype. If a parent is afflicted with the same

disease as the child, then a dominant allele is the most likely causal and a single heterozygous variant is enough for disease exhibition.

When considering two heterozygous variants within the patient, the primary question is whether they contribute together to the exhibition of a disease phenotype. To this end, let us consider all conceivable parental genotype constellations and their subsequent effect on patient compound heterozygosity and possible pathogenic status. Furthermore, all cases where a patient has variants in *cis* to one another are considered to be non-pathogenic and are already marked and filtered. Thus, the question then becomes what parental genotype sequences result in a compound heterozygous patient but can still be filtered based on the circumstances. As each parent can either be homozygous reference, homozygous alternate or heterozygous, combining assumptions 5 and 6 allows us to reduce the problem to 6 primary cases (see Fig 1), as the genotype constellations can be switched between parents and between chromosomes without effect. Here, both chromosomes of parent 1, parent 2, and the child are shown, represented by the vertical black lines. The upper variant is indicated through a red x, while the lower through a blue x. The patient is always heterozygous for both variants.

The six primary cases are:

- One parent is homozygous for the reference genotype (homozygous reference), the other heterozygous
- One parent is homozygous for the variant genotype (homozygous variant), the other heterozygous
- Both parents are homozygous variant
- Both parents are homozygous reference
- Both parents are homozygous; one reference, the other variant
- Both parents are heterozygous

The first case (1.**a**) has parent 1 also being heterozygous for the first, red variant, while parent 2 is homozygous reference. This implies that the patient received the red variant containing chromosome from parent 1, and the other chromosome from parent 2. For the patient to be compound heterozygous in this case, he must receive the second variant from parent 2. The exact chromosome the variants comes from cannot be determined, as indicated by the outlined blue variant markings on parent 2. The red variant inherited from parent 1 could possibly be on the other chromosome or in parent 2, but as previously stated, using assumptions 5 and 6, this case will always be resolved with the same logic. No further information can be gained from this pattern.

The second case (1.**b**) has parent 1 being homozygous for the first variant, while parent 2 is heterozygous. This implies that the patient received the chromosome lacking the first variant from parent 2, as parent 2 is the only parent possessing a chromosome that does not contain the red variant. Following, for the patient to be compound heterozygous, this chromosome, received from

parent 2, must contain the second variant. This implies parent 2 must be compound heterozygous for these two variants. Since both parents do not show the negative phenotype as per assumption 4, the first, red variant can be labeled as *innocuous* as all variants analyzed in conjunction lie either on the same chromosome or are of no interest, even if they are compound heterozygous. In addition, considering parent 1 is homozygous for the variant and does not exhibit any negative effects, this variant cannot be deleterious.

The third case (1.**c**) shows both parents being homozygous for the first variant. A *de novo* mutation must have occurred to allow the patient to only be heterozygous here. This variant can be labeled as *innocuous* in combination with all further variants stemming from simple inheritance from a parent, i.e. not from a *de novo* mutation where both parents are homozygous reference. Because the parent from which the alternate allele was inherited must be compound heterozygous but exhibits no pathogenic phenotype as per assumption 4, this variant combination can be labeled *innocuous*.

The fourth case (1.**d**) shows both parents being homozygous for the reference genotype. A *de novo* mutation must have occurred to allow the patient to be heterozygous here. As the parents do not possess the variant at all, no additional information can be won.

The fifth case (1.**e**) has parent 1 again being homozygous for the first variant, while parent 2 is homozygous for the reference genotype. Here, the argument runs analog to the first case, not allowing the immediate labeling of the first variant as *innocuous*. If parent 1 also possesses the second, blue variant on at least one chromosome, then it can be deduced that even in compound heterozygous cases, the first variant is not disease-causing. Thus, the first variant is labeled as homozygous for parent 1, and the combination with all other variants where parent 1 exhibits at least one variant containing chromosome can be labeled as *innocuous*. Again, it can also be argued that the first, red variant is generally of little importance because even when it is shown in the homozygous state, no deleterious effect is observed.

The final and most critical case (1.**f**) is when parent 1, parent 2, and patient are heterozygous for the first, red variant. This is the well known triple heterozygous case where trio information provides no assistance in the phasing of the patient. Further analysis shows that the patient inherited the first, red variant from either parent 1 or parent 2. Thus, the second, blue variant must be inherited from the other parent and from exactly that chromosome which does not contain the first, red variant. This implies that the parent from which the second, blue variant is inherited must also be compound heterozygous. Thus, according to assumption 4, all compound heterozygous variant combinations that contain the first, red variant must be innocuous. This implies that all potentially damaging compound heterozygous locations within a genome can be determined if all variants have been successfully called in the patient and both parents. As all other cases allow trio phasing to say with certainty from which parent each variant was inherited, the ability to label triple heterozygous patterns as *innocuous* resolves the last bastion of uncertainty for the clinical analysis of compound heterozygous variants, provided all variants have been successfully and accurately called.

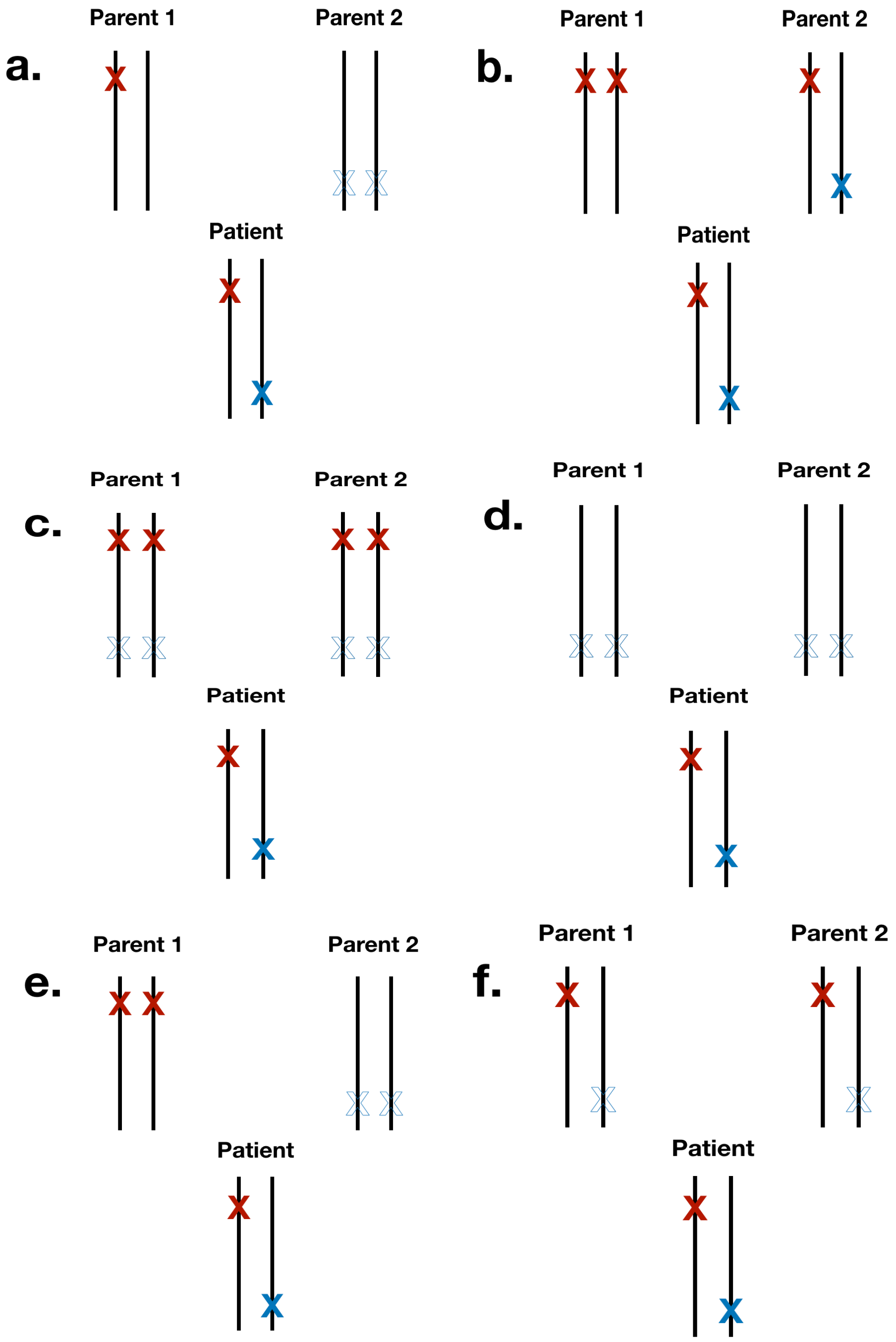

Figure 1: **Innocuous labeling.** Possible inheritance patterns that produce compound heterozygosity in the patient. The hollow blue Xs show possible parent variant positions that would result in the child's genotype.

1.4 Bitflag system

For each variant pair that is processed by SmartPhase, a flag is given that provides details of how a pair was phased or why it could not be phased. To capture the full complexity, we introduced five flags indicating whether a pair was phased as *cis*, *trans*, not phased, labeled as *innocuous* or not found. The first bit is set if the variant pair lies on the same haplotype (*cis*). The second bit is set if the pair is compound heterozygous (*trans*). The third bit is set if the variant pair is not phased due to lack of evidence. The fourth bit is set if the variant pair is *innocuous*. The fifth bit is set if the variant pair contains a variant that was not found in the given read alignment of the provided mapping files. Table A shows the different possible combinations of flags and the resulting final flag. Variant pairs may be phased even if one or both variants are not found either by trio phasing or by using GATK physical phasing information.

Final flag	*Cis*	*Trans*	Not phased	*Innocuous*	Not found
1	x				
2		x			
4			x		
9	x			x	
10		x		x	
12			x	x	
17	x				x
18		x			x
20			x		x
25	x			x	x
26		x		x	x
28			x	x	x

Table A: **List of all possible bit flag combinations.**

2 Validation on Simulated WES Data

2.1 Simulation of WES data

To accurately validate SmartPhase a simulated data set with known phase was generated. The widely used CEU (NA12878, NA12891, NA12892) and YRI (NA19238, NA19239, NA19240) trio data sets were selected to produce simulated data. The consensus genotype call sets for both data

sets were taken from the 1000 Genomes Project [6] (20140625_high_coverage_trios_broad). For each data set, SHAPEIT (v2.r837) was used to create a base phase of all variants of the patients [7]. An artificial child and its simulated genome based on the phased parents were created analogously to the validation of the phasing tool WhatsHap [8]. Wessim was used to perform *in silico* exome capture based on the probe sequences of the SureSelect Human All Exon V6+UTR Kit (Agilent Technologies) [9]. Using the error model of an Illumina Genome Analyzer IIx with TrueSeq SBS Kit v5-GA provided by GemSim [10], we generated paired-end reads of length 100 with an average coverage of 126 across the captured regions of chromosome 1 and 19. These reads were then mapped to the human reference genome GRCh37 using BWA-MEM (v0.7.15) [11]. The genotype call sets obtained from the 1000 Genomes Project and the subsequent generated BAM files were used to benchmark SmartPhase and WhatsHap.

2.2 Selection of candidate variant pairs

We selected all genes that harbor less than 10 heterozygous variants according to the reference variant calls of the 1000 Genomes Project [6] in order to prevent genes with multiple heterozygous variants from dominating the evaluation as the number of potential pairs grows exponentially with the number of variants. This is done as an overabundance of proximal variants is particularly easy to phase and would unrealistically inflate the benchmarks. Additionally, those genes that did not contain at least two heterozygous variants were not phased as these cannot be affected by compound heterozygosity. As a result, we generated a set of $26,638$ potential heterozygous variant pairs distributed over $2,922$ genes with 4.21 heterozygous variants per gene on average (see Table B).

Trio	Chr	Genes	Variants	Pairs
CEU	1	748	$3,139$	$6,783$
CEU	19	528	$2,153$	$4,531$
YRI	1	939	$4,081$	$9,186$
YRI	19	707	$2,933$	$6,138$

Table B: **Overview over the number of genes and variants in the simulation data set.** The number of genes is different for both trios because genes with more than 10 heterozygous variants were removed from the benchmark. For the CEU trio, 67 genes on chromosome 1 and 46 genes on chromosome 19 were excluded for having too many variants. For the YRI trio, 102 genes on chromosome 1 and 65 genes on chromosome 19 were removed for having too many variants.

2.3 Configuration of SmartPhase and WhatsHap

WhatsHap is a software that implements a read-based phasing algorithm leveraging long-reads generated by the latest sequencing technologies [12]. The algorithm is further able to incorporate sequencing information of related individuals [13]. We used the WhatsHap tool (v0.17) [8] to

phase genomic variants within genes of our simulated WES data. To ensure comparability, we reduced the variant files and simulated mapping files to regions within protein-coding genes as defined by the canonical gene regions of the GRCh37 assembly of the UCSC genes track returned by the UCSC table browser [14].

SmartPhase and WhatsHap were both configured to only use reads with a maximum mapping quality of 60 and phase single nucleotide variants as well as insertions and deletions. For trio phasing, we provided the genomic variants of the parents as given by the 1000 Genomes Project [6] as additional input.

3 Validation on Clinical WES Data

3.1 Processing of WES data

We used WES data generated at the Care-for-Rare Genomics Core Facility Laboratories at the Dr. von Hauner Children's Hospital. We selected a cohort of $1,163$ individuals consisting of 921 patients suffering from rare immunological pediatric diseases and 242 healthy parents. Genomic DNA of the samples was isolated using the QIAmp DNA Blood Mini Kit (Qiagen) according to the manufacturer's instructions. After enriching for coding exons using the SureSelect Human All Exon V6+UTR Kit (Agilent Technologies), sequencing was performed on an Illumina NextSeq 500 system. BWA-MEM (v0.7.15) [11] was used to map the short paired reads to the human reference genome GRCh37. The Genome Analysis Toolkit (GATK v3.8) was used for joint variant calling according to best practices [15, 16]. To reduce the number of false-positive variant calls we filtered individual genotypes by their DP and GQ values [17]. Variant Effect Predictor based on Ensembl release 92 [18] and GEMINI (v0.20.1) were used to create a database of variants.

3.2 Selection of candidate variant pairs

Potentially pathogenic compound heterozygous variant pairs were determined by selecting all autosomal heterozygous variants in patients that have a predicted impact on the amino acid sequence. Variants were excluded if their allele frequency was larger than 10% within the cohort of 921 patients and 242 healthy parents. A maximum allele frequency threshold of 5% using the 1000 Genomes Project [6] and 0.5% using the exomes of the gnomAD database [19] was applied to further reduce the set of variants. The gnomAD database was further used to filter out variant positions where the alternate allele has been observed as a homozygous genotype at least once in the set of exomes. The final set of $116,613$ potential compound heterozygous variant pairs was generated by computing all possible combinations of the remaining variants per gene for each patient.

3.3 Validation approaches

The clinical WES data set was used to perform two different validations. First, we evaluated the clinical utility of SmartPhase on all candidate variant pairs by comparing its ability to exclude

non-pathogenic variant combinations in singleton and trio patients with or without incorporating physical phasing information. Second, we compared the performance of SmartPhase and WhatsHap on all trio patients in *read only* and combined *read & trio* phasing mode.

3.3.1 Overall performance of SmartPhase

The performance of SmartPhase is evaluated on the complete set of $116,613$ potential compound heterozygous variant pairs identified in the 800 singleton and 121 trio patients of the cohort. SmartPhase was configured to use parental genotype information for the trio patients. It was executed twice to compare the performance with and without the use of physical phasing information. A detailed description of the results can be found in the manuscript in the corresponding section. S2 Table provides all relevant numbers.

3.3.2 Comparison of SmartPhase to WhatsHap

The comparison is based on all $21,066$ candidate variant pairs identified in the 121 trio patients to compare the performance of *read only* and combined *read & trio* phasing. Analogous to the comparison on simulated data, SmartPhase and WhatsHap were both configured to only use reads with a maximum mapping quality of 60 and phase single nucleotide variants as well as insertions and deletions. Both SmartPhase and WhatsHap were applied to the data set in *read only* and combined *read & trio* mode with SmartPhase using provided physical phasing information. The results are described in the corresponding paragraph of the manuscript and supplemented by a detailed list of all numbers in S3 Table.

References

[1] Choi K, Henderson IR. Meiotic recombination hotspots – a comparative view. The Plant Journal. 2015;83(1):52–61. doi:10.1111/tpj.12870.

[2] Myers S, Bottolo L, Freeman C, McVean G, Donnelly P. A Fine-Scale Map of Recombination Rates and Hotspots Across the Human Genome. Science. 2005;310(5746):321–324. doi:10.1126/science.1117196.

[3] Jiang Y, McCarthy JM, Allen AS. Testing the Effect of Rare Compound-Heterozygous and Recessive Mutations in Case-Parent Sequencing Studies. Genetic Epidemiology. 2015;39(3):166–172. doi:10.1002/gepi.21885.

[4] Yu T, Chahrour M, Coulter M, Jiralerspong S, Okamura-Ikeda K, Ataman B, et al. Using whole exome sequencing to identify inherited causes of autism. Neuron. 2013;77(2):259–273. doi:10.1016/j.neuron.2012.11.002.

[5] Enomoto A, Kimura H, Chairoungdua A, Shigeta Y, Jutabha P, Ho Cha S, et al. Molecular identification of a renal urate-anion exchanger that regulates blood urate levels. Nature. 2002;417(6887):447–452.

[6] Auton A, Abecasis GR, Altshuler DM, Durbin RM, Abecasis GR, Bentley DR, et al. A global reference for human genetic variation. Nature. 2015;526(7571):68–74. doi:10.1038/nature15393.

[7] Delaneau O, Marchini J, Zagury JF. A linear complexity phasing method for thousands of genomes. Nature Methods. 2012;9(2):179–181. doi:10.1038/nmeth.1785.

[8] Martin M, Patterson M, Garg S, Fischer SO, Pisanti N, Klau GW, et al. WhatsHap: fast and accurate read-based phasing. bioRxiv. 2016; p. 085050. doi:10.1101/085050.

[9] Kim S, Jeong K, Bafna V. Wessim: a whole-exome sequencing simulator based on in silico exome capture. Bioinformatics. 2013;29(8):1076. doi:10.1093/BIOINFORMATICS/BTT074.

[10] McElroy KE, Luciani F, Thomas T. GemSIM: general, error-model based simulator of next-generation sequencing data. BMC Genomics. 2012;13(1):74. doi:10.1186/1471-2164-13-74.

[11] Li H. Aligning sequence reads, clone sequences and assembly contigs with BWA-MEM; 2013. arXiv:1303.3997v2 [q-bio.GN]. Available from: http://arxiv.org/abs/1303.3997.

[12] Patterson M, Marschall T, Pisanti N, van Iersel L, Stougie L, Klau GW, et al. W hats H ap : Weighted Haplotype Assembly for Future-Generation Sequencing Reads. Journal of Computational Biology. 2015;22(6):498–509. doi:10.1089/cmb.2014.0157.

[13] Garg S, Martin M, Marschall T. Read-based phasing of related individuals. Bioinformatics. 2016;32(12):i234–i242. doi:10.1093/bioinformatics/btw276.

[14] Karolchik D, Hinrichs AS, Furey TS, Roskin KM, Sugnet CW, Haussler D, et al. The UCSC Table Browser data retrieval tool. Nucleic acids research. 2004;32(suppl_1):D493–D496.

[15] Van der Auwera GA, Carneiro MO, Hartl C, Poplin R, del Angel G, Levy-Moonshine A, et al. From FastQ Data to High-Confidence Variant Calls: The Genome Analysis Toolkit Best Practices Pipeline. In: Current Protocols in Bioinformatics. vol. 43. Hoboken, NJ, USA: John Wiley & Sons, Inc.; 2013. p. 11.10.1–11.10.33. Available from: http://www.ncbi.nlm.nih.gov/pubmed/25431634http://www.pubmedcentral.nih.gov/articlerender.fcgi?artid=PMC4243306http://doi.wiley.com/10.1002/0471250953.bi1110s43.

[16] Poplin R, Ruano-Rubio V, DePristo MA, Fennell TJ, Carneiro MO, der Auwera GAV, et al. Scaling accurate genetic variant discovery to tens of thousands of samples. bioRxiv. 2018; p. 201178. doi:10.1101/201178.

[17] Carson AR, Smith EN, Matsui H, Brækkan SK, Jepsen K, Hansen JB, et al. Effective filtering strategies to improve data quality from population-based whole exome sequencing studies. BMC Nephrology. 2014;15(1):125. doi:10.1186/1471-2105-15-125.

[18] McLaren W, Gil L, Hunt SE, Riat HS, Ritchie GRS, Thormann A, et al. The Ensembl Variant Effect Predictor. Genome biology. 2016;17(1):122. doi:10.1186/s13059-016-0974-4.

[19] Lek M, Karczewski KJ, Minikel EV, Samocha KE, Banks E, Fennell T, et al. Analysis of protein-coding genetic variation in 60,706 humans. Nature. 2016;536(7616):285–291. doi:10.1038/nature19057.
