# Supplementary material for: SmartPhase: Accurate and fast phasing of heterozygous variant pairs for genetic diagnosis of rare diseases
Source: PLoS Comput Biol. 2020 Feb 7;16(2):e1007613. doi: 10.1371/journal.pcbi.1007613 (PMC7032729; doi:10.1371/journal.pcbi.1007613)
Supplement: S1 Text — The documentation gives instructions on how to run SmartPhase and how to interpret its results. (PDF) [file pcbi.1007613.s006.pdf]

SmartPhase

SmartPhase is a phasing tool tailored for clinical use in genetic diagnosis pipelines. It accurately and efficiently reduces the number of possible compound heterozygous variant pairs being examined around either predefined genetic loci or from a list of preselected variant pairs. To achieve this, SmartPhase is able to incorporate parental genotype information as well as reads generated from DNA- or RNA-sequencing. Furthermore, it incorporates existing haplotype information and applies logical rules to exclude variant constellations that cannot be disease causing.

For a more thorough explanation of SmartPhase and its validation, please refer to the following:

SmartPhase is offered as an executable JAR, but can also be compiled from its source code. The following documentation serves as a general overview and explanation of all its arguments and recommended values.

Availability

You can download the published version of SmartPhase here. After extracting the archive you can use it as you would have cloned it from the GitHub respository. In our GitHub respository we provide the latest updates on SmartPhase. The data simulated for the validation of SmartPhase can be downloaded here.

Requirements

- Java 10

Compiling

If you want to compile SmartPhase from source execute the following commands

```
git clone https://github.com/paulhager/smart-phase.git
cd ./smart-phase/
bash compile.sh
```

The resulting `smartPhase.jar` file will be located in `./compile/smartPhase.jar`. This can then be moved one folder up to replace the included pre-compiled JAR. If you do so, you can immediately test SmartPhase with the Use Case command explained below. Otherwise, you have to adjust for the location of the `smartPhase.jar` accordingly.

SmartPhase can also be built using maven. To do so, execute the following commands

```
git clone https://github.com/paulhager/smart-phase.git
cd ./smart-phase/Phase/src/
mvn clean install
```

The resulting `smartPhase-x.x.x.jar` file will be located in `./Phase/src/target/smartPhase-x.x.x.jar`. This can then be moved to the repository root directory to replace the included pre-compiled JAR. If you do so, you can immediately test SmartPhase with the Use Case command explained below. Otherwise, you have to adjust for the location of the `smartPhase-x.x.x.jar` accordingly.

Use Case

After cloning the repository, you can directly run SmartPhase on a Use Case scenario by executing the following command from the `smart-phase` folder. If you have compiled the `smartPhase.jar` file yourself as described above you have to adjust the path to it accordingly. The results will be written into the `UseCase` folder.

```
java -jar smartPhase.jar -a ./UseCase/CEU_UseCase.vcf.gz -p NA12878 \
-g ./BED/allGeneRegionsCanonical.HG19.GRCh37.bed \
-r ./UseCase/CEU_UseCase.bam -m 60 \
-d ./UseCase/CEU.ped -o ./UseCase/CEU_UseCase_results.tsv \
-x -t -vcf -c 0.1
```

Details on the underlying data and the generated results are described in the corresponding README.

Running SmartPhase

SmartPhase can either be run in *explorative* or *analytic/paired* mode. In *explorative* mode, SmartPhase parses provided genomic regions of interest (typically protein coding regions) provided in a BED file and, per region, phases either all variants or merely those specified in a filtered variants file. In *analytic* or *paired* mode, no regions of interest must be given, just specific variant pairs that should be phased to one another have to be provided. SmartPhase will then create the appropriate regions. The *analytic* mode lends itself more to an analysis of a cohort where pre-filtering has already been done and only specific candidate variant pairs should be analyzed whereas the *explorative* mode is more all-purpose and can be run at any point in the analysis pipeline.

Basic usage

```
java -jar SmartPhase.jar \
```

```
-g /path/to/genomic/regions/to/be/phased/allGeneRegionsCanonical.HG19.GRCh37.bed \
-a /path/to/vcf/containing/all/variants/sample.vcf.gz -p PID12345 \
-r /path/to/bam/containing/reads/DNAseq.bam,/path/to/bam/containing/reads/RNAseq.bam \
-m 60,255 \
-d /path/to/ped/family.ped -t -o /path/to/desired/output/file/output.tsv
```

Options

`-a or --all-variants`

REQUIRED. The path to the VCF file containing all variants. This is usually set to the VCF file generated at the end of the variant calling and filtering pipeline. To only phase selected variants here use a combination of the genomic intervals and filtered variants arguments (-f and -g).

`-p or --patient`

REQUIRED. The sample identifier used to refer to the patient of interest in the VCF and PED files. Must be internally consistent throughout all files.

`-o or --output`

REQUIRED. The path to the filename where the output should be written to. If the file already exists, it will be deleted on program start. The output file will be in tab seperated format.

`-f or --filtered-variants`

The path to the file containing prefiltered variants that should be phased. If running in *explorative* mode, this can be left blank and will be set equal to the all variants (-a) file provided. SmartPhase then assumes that all variants that fall within the genomic regions (-g) of interest should be phased.

If running in *analytic* or *paired* mode, this file is **REQUIRED**. In *analytic* mode variants provided here can either be in the form of a VCF file or merely a list of variants with an appropriate first-line header specifying which column contains the contig, the start position, the reference call and the alternate call. Only tab or comma seperated files of this type are accepted. A common data source for this type of file would for example be the output of a GEMINI database query.

In *paired* mode a multi-column file of pre-selected potential compound heterozygous variant pairs can be provided here. The first column may contain multiple comma seperated patient IDs. Only those lines with the patient ID specified in the patient flag (-p) will be phased. If multiple, comma seperated patient

IDs are present, the file must be tab seperated. The second and third columns are the two variants whose phase should be determined. Each variant entry must follow the following pattern `contig-start-reference-alternate`. For example: `chr1-143555-G-A`.

A full entry in such a file could look like this: `PID12345,4-722294-G-A,4-722315-T-C`

or like this: `PID12345,PID9734,PID2356 4-722294-G-A 4-722315-T-C`

`-g or --gene-regions`

The path to the BED file containing the genomic regions of interest that should be phased. Only variants within these regions will be phased. Must be in standard BED format. If this argument is not set, the *analytic/paired* mode is assumed and genomic regions will be built around the variants pairs specified in the filtered variants (-f) file.

`-r or --reads`

A comma seperated list of paths pointing to the BAM files containing the reads to be used for read backed phasing. Can either be reads generated through DNAseq or RNAseq experiments. If this option is used, the mapping quality filter option (-m) must also be used and have the same number of items. The order must be the same also. This option and/or trio backed phasing (-t) must be activated.

`-m or --mapq`

A comma seperated list of integers indicating the cut-off mapping quality value for reads in the respective file given in the reads flag (-r). For example, if the arguments passed to -r are file1,file2 and the arguments passed to -m are 255,60 this would indicate that only those reads in file1 with a mapping quality of at least 255 are to be considered and only those reads with a mapping quality of at least 60 in file2 are to be considered (in this case most likely because file1 contains reads generated through RNAseq and file2 through DNAseq and only high-confidence, uniquely mapped reads should be used).

`-t or --trio`

A boolean flag indicating that trio phasing should be done. All variant call information for mother, father and child (patient) must be given in the all variants file (-a). NOTE: If this is present, pedigree information must also be provided (-d).

`-d or --ped`

The path to the PED file containing information on the familial structure of the trio wishing to be phased. Must be a valid PED file. May contain other families as well as a long as the patient is also present.

`-y or --physical-phasing`

Indicates GATK physical phasing information present in the all variants VCF file (-a) should be used as a last resort to phase when no trio or read-backed evidences were available. If using GATK HaplotypeCaller (HC) to call variants, this information should already be present. These variants are most likely the result of local haplotype restructuring and thus are usually not able to be phased using read-backed phasing. In this case, using the information written by GATK HC during restructuring allows SmartPhase to still ascertain phase.

`-x or --reject-phase`

A boolean flag indicating that any phase information already annotated in the VCF, for example from other phasing programs, should be ignored. By default this information is taken as correct and those variants that have already been phased are not phased again. To phase all variants in a file, regardless of other phasing information present, add this argument.

`-vcf`

A boolean flag indicating that the results of smart phase should also be written to a vcf file. The file will be based on the all variants file (-a) and will contain two new info fields, SPGT and SPID. Their significance is explained the output section bellow. NOTE: If this flag is specified, a cutoff (-c) must be provided.

`-c or --cutoff`

The cutoff value to be used when deciding which phased blocks should be written in the final vcf file. If a block contains at least one variant pair with a confidence below the cutoff, the entire block is discarded. NOTE: If this argument is used, the -vcf flag must also be specified.

`-h or --help`

Print a summary of the above explanations.

Output

General Specification

The default output generated by SmartPhase is stored in a tab-seperated format and consists of 5 columns.

A typical line in the output file would look like this:

```
C1orf170-1-910578-917473    1-914333-C-G    1-914521-C-T    2   0.75
```

The first column is the label of the region this variant pair fell into in the format: `name-contig-start-stop`. The second column shows the first variant in the pair in standard variant format: `contig-start-reference-alternate`. The third column shows the second variant in the pair, also in standard variant format. The fourth column is the flag representing the phasing information determined during execution (see the next section for an explanation of the flags). And the fifth column is a confidence score indicating how confident SmartPhase is in its phasing call. If a variant pair was phased using trio information, the confidence is set to 1. If read information was used, the confidence takes into account how much conflicting evidence was found and the total number of reads examined. A more thorough explanation of the confidence system can be found in our paper.

When the -vcf flag is specified, a bgzipped vcf will also be generated where the haplotype blocks of the individual variants and their phase are recorded. This is done with the SPGT and SPID genotype format fields. SPGT specifies the genotype or phase of the variant with respect to the other variants in the block. SPID identifies the block the variant belongs to and is made up of the first variant in the blocks start position and its chromosome. If a block contains at least one variant pair with a confidence below the cutoff (specified in -c), the entire block is discarded.

Flags

Bit	Cis	Trans	Not phased	Innocuous	Not found
1	x				
2		x			
4			x		
9	x			x	
10		x		x	
12			x	x	

Bit	Cis	Trans	Not phased	Innocuous	Not found
17	x				x
18		x			x
20			x		x
25	x			x	x
26		x		x	x
28			x	x	x
